# Thermal Decomposition Properties of Epoxy Resin in SF_6_/N_2_ Mixture

**DOI:** 10.3390/ma12010075

**Published:** 2018-12-26

**Authors:** Hao Wen, Xiaoxing Zhang, Rong Xia, Zilai Yang, Yunjian Wu

**Affiliations:** 1 School of Electrical Engineering, Wuhan University, Wuhan 430072, China; wenhao198711@163.com (H.W.); yangzilai123@163.com (Z.Y.); wuyunjian@whu.edu.cn (Y.W.); 2 Wuhan Branch, China Electric Power Research Institute Co., Ltd., Wuhan 430074, China; xiarong@epri.sgcc.com.cn

**Keywords:** SF_6_/N_2_, thermal decomposition, epoxy resin, decomposition components

## Abstract

As a promising alternative for pure SF_6_, the mixture of SF_6_/N_2_ appears to be more economic and environment-friendly on the premise of maintaining similar dielectric properties with pure SF_6_. But less attention has been paid to the thermal properties of an SF_6_/N_2_ mixture, especially with insulation materials overheating happening simultaneously. In this paper, thermal decomposition properties of epoxy resin in SF_6_/N_2_ mixture with different SF_6_ volume rates were studied, and the concentrations of characteristic decomposition components were detected based on concentrations change of some characteristic gas components such as CO_2_, SO_2_, H_2_S, SOF_2_, and CF_4_. The results showed that thermal properties of 20% SF_6_/N_2_ (volume fraction of SF_6_ is 20%) mixture has faster degradation than 40% SF_6_/N_2_ mixture. As ratio of SF_6_ content decreases, thermal stability of the system decreases, and the decomposition process of SF_6_ is exacerbated. Moreover, a mathematical model was established to determine happening of partial overheating faults on the epoxy resin surface in SF_6_/N_2_ mixture. Also thermal decomposition process of epoxy resin was simulated by the ReaxFF force field to reveal basic chemical reactions in terms of bond-breaking order, which further verified that CO_2_ and H_2_O produced during thermal decomposition of epoxy resin can intensify degradation of SF_6_ dielectric property.

## 1. Introduction

SF_6_ gas is colorless, odorless and nontoxic, and is widely used as power equipment’s insulation material because of its excellent arc-extinguishing and insulating properties [1,2,3,4,5,6,7]. Its chemical property is so stable that it can stably remain in the atmosphere for 2300 years, and its global warming potential (GWP) is 2500 times that of CO_2_. Hence, it was listed as one of the six greenhouse gases in Kyoto Protocol in 1997. To reduce the use of SF_6_, researchers worldwide have developed new alternatives such as C_4_F_7_N, C_5_F_10_O, SF_6_/CO_2_, and SF_6_/N_2_ mixture as insulation gases and put them into practice [3,5,8,9,10]. Among these gases, an SF_6_/N_2_ mixture with low SF_6_ volume fraction has been widely used in electric equipment such as gas insulated transmission lines (GIL) [6,11,12,13,14]. In the 1970s, 420/550 kV transmission lines of 20% SF_6_/N_2_ (volume fraction of SF_6_ is 20%) were developed by SIMENS and put into use in Geneva, Switzerland, which proved to be of high economic benefits. The transmission lines of 10% SF_6_/N_2_ gases in Electricite De France (EDF) have been safely used up to the present.

When there happens to be an overloading problem in GIL, partial overheating failure is likely to occur at the insulation defects spots, especially in the poor contact surface, which would do harm to the dielectric properties of the insulation material, and the defects will aggravate partial overheating failure in return. The deterioration of insulation material will lead to serious consequences, such as the failure of some key part, or even a blackout at worst [15,16,17,18]. As a widely used insulation material, epoxy resin is often used together with SF_6_ in electrical equipment, such as supporting spacers in GIL. In order to detect partial overheating failure as early as possible, it has been proposed to detect the thermal decomposition components of SF_6_ (such as SO_2_, H_2_S, SOF_2_, SO_2_F_2_, CF_4_, CO_2_, and SOF_4_) in the presence of organic solid insulation materials [2,16,19]. When partial overheating failure happens temperature also can be estimated approximately based on the concentration change of the components. N_2_ molecules in the SF_6_/N_2_ mixture will make SF_6_ molecules burden uneven stress distribution, causing increase in the bonding energy of S-F [20,21,22,23,24]. The increase of intramolecular energy hinders system stability, which intensifies the impact of partial overheating. As an organic insulating material, the decomposition products of epoxy resin will also affect the development of partial overheating. The method for detecting thermal decomposition components in SF_6_/N_2_ mixture in the presence of epoxy resin has not been reported yet. Hence, this issue has been investigated in this paper.

Thermogravimetric analysis instrument was used to investigate the decomposition of epoxy resin in SF_6_/N_2_ mixture with SF_6_ ratio of 20%, 30%, and 40% respectively (20% SF_6_/N_2_, 30% SF_6_/N_2_, and 40% SF_6_/N_2_ in short). By analyzing TG/DSC curves and comparing them with those from epoxy resin decomposition under pure SF_6_ or N_2_, the effect of ratio of SF_6_ on the decomposition of epoxy resin was obtained. The variation of SF_6_ characteristic decomposition component concentration with temperature in three different proportions of mixed gases was observed by Shimadzu QP2010 Ultra GC/MS. Types of gases that could be used as the characteristic components to detect the partial overheating failure in the presence of epoxy resin have been determined. Also the criterion of sharp weight loss of epoxy resin is established based on the change of characteristic components ratio.

## 2. Materials and Methods

### 2.1. Materials

In the experiment, the bisphenol-A epoxy resin E51 was purchased from Wuxi Lan-Star Petrochemical Co., Ltd. (Wuxi, China). Epoxy resin was cured by an amine curing agent dubbed as type 593, supplied by Wuhan Shen Chemical Reagents and Equipments Co., Ltd. (Wuhan, China). High-precision SF_6_/N_2_ mixture gas was supplied by Wuhan Newradar Gas Co., Ltd. (Wuhan, China). The mixture gas was prepared according to the reference material number GBW(E)061516 in China, so the original percentage content of O_2_ and H_2_O in the mixture gas were too low to be took into consideration. Therefore, the main resource of H_2_O in the heating process may come from the thermal decomposition of epoxy resin.

### 2.2. Parameters in the Experiment

In order to obtain the TG/DSC curves of epoxy resin decomposition in SF_6_/N_2_ mixture, TGA/DSC 3+ thermogravimetric analyzer of Mettler Toledo company was used in the experiment. At first three parameters including thermal conductivity, density and specific heat capacity were calculated in order to keep the accuracy of experiments after filling them in the sheet of parameters of TG/DSC instrument.

#### 2.2.1. Density of SF_6_/N_2_ Gas Mixture

The instrument uses gas density at 0 °C under standard atmospheric pressure. The density of SF_6_/N_2_ mixture is given by:

(1) $$\rho_{0}=\frac{\rho_{1}\rho_{2}}{\rho_{2}m_{1}+\rho_{1}m_{2}}$$

where $\rho_{1}$ is the density of SF_6_; $\rho_{2}$ is the density of N_2_, $m_{1}$ and $m_{2}$ are mass percentages of SF_6_ and N_2_ respectively in per mole of mixture. The calculation results are shown in Table 1.

#### 2.2.2. Specific Heat Capacity of SF_6_/N_2_ Gas Mixture

The instrument uses heat capacity at 25 °C under standard atmospheric pressure. The heat capacity of SF_6_/N_2_ gas mixture is calculated as follows:

(2) $$C_{P}=C_{P1}*m_{1}+C_{P2}*m_{2}$$

where $C_{P1}$ and $C_{P2}$ represent the specific heat capacity of SF_6_ and N_2_ gas under constant pressure respectively; $m_{1}$ and $m_{2}$ are mass percentages of SF_6_ and N_2_ in per mole of mixture respectively. The calculation results are shown in Table 2.

#### 2.2.3. Thermal Conductivity of SF_6_/N_2_ Gas Mixture

The instrument uses thermal conductivity at 0 °C under standard atmospheric pressure. The thermal conductivity of SF_6_/N_2_ gas mixture λ is given by:

(3) $$\lambda =\frac{\lambda_{1}y_{1}}{y_{1}+A_{12}y_{2}}+\frac{\lambda_{2}y_{2}}{y_{2}+A_{21}y_{1}}$$

where $\lambda_{1}$ and $\lambda_{2}$ represent the thermal conductivity of SF_6_ and N_2_ under atmospheric pressure, $y_{1}$ and $y_{2}$ are the molar fractions of SF_6_ and N_2_ in the mixture respectively, $A_{12}$ and $A_{21}$ are constants given by:

(4) $$A_{12}=\frac{1}{4}{\left\{1+{\left[\left(\frac{u_{1}}{u_{2}}\right){\left(\frac{M_{2}}{M_{1}}\right)}^{\frac{3}{4}}\left(\frac{1+\frac{1.5T_{b1}}{T}}{1+\frac{1.5T_{b2}}{T}}\right)\right]}^{\frac{1}{2}}\right\}}^{2}\frac{\left(1+\frac{S_{12}}{T}\right)}{\left(1+\frac{1.5T_{b1}}{T}\right)}$$

(5) $$A_{12}=\frac{1}{4}{\left\{1+{\left[\left(\frac{u_{1}}{u_{2}}\right){\left(\frac{M_{2}}{M_{1}}\right)}^{\frac{3}{4}}\left(\frac{1+\frac{1.5T_{b1}}{T}}{1+\frac{1.5T_{b2}}{T}}\right)\right]}^{\frac{1}{2}}\right\}}^{2}\frac{\left(1+\frac{S_{12}}{T}\right)}{\left(1+\frac{1.5T_{b1}}{T}\right)}$$

where $u_{1}$ and $u_{2}$ represent the viscosity of SF_6_ and N_2_ under atmospheric pressure (kg·s/m^2^), $M_{1}$ and $M_{2}$ are relative mass fractions of SF_6_ and N_2_, $T_{b1}$ and $T_{b2}$ are the boiling points of SF_6_ and N_2_ under atmospheric pressure (K), T is the constant of 273.15 (K), $S_{12}$ and $S_{21}$ are given by:

(6) $$S_{12}=S_{21}=\sqrt{2.25T_{b1}T_{b2}}$$

The calculated values of thermal conductivities are shown in Table 3.

### 2.3. Experiment Process

In this paper, SF_6_/N_2_ mixture gas with the SF_6_ volume ratio from 20% to 40% were used in experiment, and the results were compared with those from pure SF_6_ and N_2_. The sample mass of cured epoxy resin was 20 mg. The gas flow rate was set as 20 mL/min, the heating rate as 10 °C/min heating in the temperature range from 250 °C to 650 °C.

## 3. Results and Discussion

### 3.1. TG Curve Analysis of Epoxy Resin Decomposition

**Figure 1:**
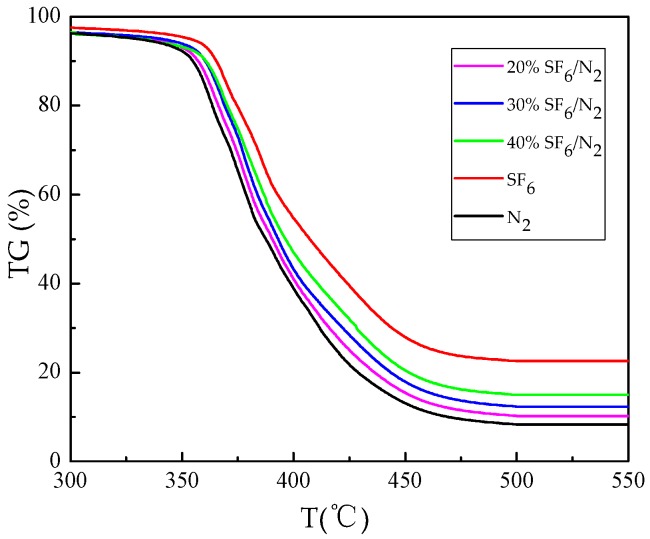
TG curve of epoxy resin decomposition under different experimental gases.

Figure 1 shows the TG curves of epoxy resin decomposed under different experimental gases. As can be seen that the main weight loss of epoxy resin happened in the temperature range from 330 °C to 470 °C, which are not affected by the type of gas in reaction. But it is noteworthy that the weight-loss ratio of epoxy resin varied with the type of gas in reaction, such as under N_2_ condition, the decomposition of epoxy resin is most violent, with remaining mass of 1.59 mg, accounting for 7.97% of the total weight of experimental sample, while under SF_6_ condition, the amount of remaining mass of decomposed epoxy resin was 4.53 mg, accounting for 22.73% of the total weight of experimental sample. The decomposition extent of epoxy resin in three mixed gases follows: 20% SF_6_/N_2_ > 30% SF_6_/N_2_ > 40% SF_6_/N_2_, that is to say, in the mixture of 20% SF_6_/N_2_, weight loss of epoxy resin was more severe than in the mixture of 40% SF_6_/N_2_.

As the reaction gas, thermal conductivity of SF_6_ is lower than that of N_2_, therefore, the poor thermal conductivity of SF_6_ is not conducive to the decomposition of epoxy resin. However, N_2_ has higher thermal stability due to its large molecular bond energy of 946 kJ/mol. So N_2_ will not decompose or react with epoxy resin in the experimental temperature range. In pure SF_6_, SF_6_ begins to decompose at about 260 °C, and reaction between SF_6_ and epoxy resin would help add to the whole weight of the residue. The decrease of SF_6_ content in gas mixture will reduce the thermal stability of the system, making epoxy resin easier to react with SF_6_.

### 3.2. DSC Curve Analysis of Epoxy Resin Decomposition

**Figure 2:**
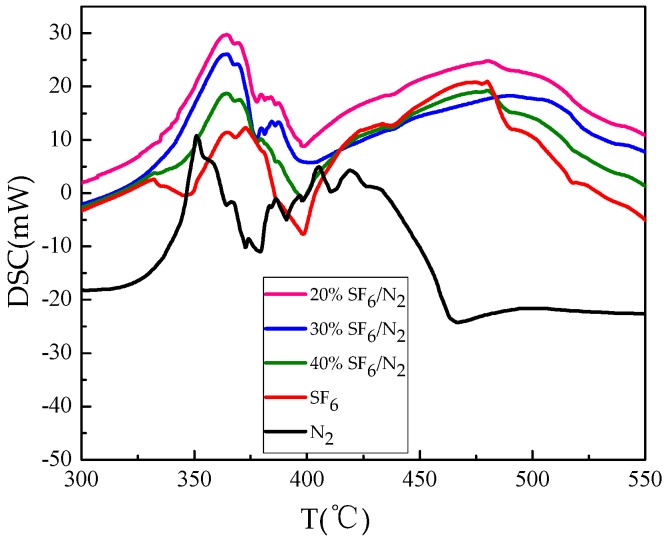
DSC curve of epoxy resin decomposed under different experimental gas conditions.

In the DSC curve, the convex peak represents an increase in enthalpy(exothermic) and the concave peak represents a decrease in enthalpy(endotherm). Figure 2 is the DSC curves of epoxy resin decomposed under different experimental gas conditions. As can be seen from Figure 2, the decomposition process of epoxy resin is complicated. Within the main weight loss range of 340 °C–470 °C, there are obvious characteristic peaks of heat absorption and release. The process is divided into three reaction stages: melting, exothermic behavior (solidification, oxidation, reaction, crosslinking), decomposition, and gasification. The peak area of heat absorption indicates the reaction energy level. By integrating the endothermic peak area in the main weight loss interval, the endothermic peak energy is obtained and shown in Table 4. The value arranged from big to small in the following order: 20% SF_6_/N_2_ > 30% SF_6_/N_2_ > 40% SF_6_/N_2_ > SF_6_ > N_2_.

During the exothermic behavior stage, the peak temperature at this stage is greatly affected by SF_6_. In pure SF_6_, the temperature at the exothermic peak is about 370 °C versus 350 °C in pure N_2_. Besides, in the pure N_2_, around 400 °C there are also existing some exothermic peaks representing the intrinsic thermal decomposition of epoxy resin. With the amount of SF_6_ increasing, some consecutive small peaks showed up at about 385 °C. Thermal decomposition of epoxy resin is actually the process of breaking and regenerating chemical bonds. In epoxy resin, C-O bond and C-H bond account for the major chemical bonds, and they are easy to break due to low bond energy. After breaking, free C, H and O atoms are formed, and further combined to form small molecules such as H_2_O and CO_2_. As a highly thermal stable gas, chemical bonds in N_2_ do not break to form N atoms under experimental temperature conditions, therefore, epoxy resin has intrinsic bond breakage in N_2_. SF_6_ decomposes at 260 °C in the presence of organic insulating solids [2]. And SF_6_ decomposes continuously throughout the weight-losing temperature range, a continuous exothermic peaks were shown on the DSC curve, causing the peak values to shift to the high temperature zone compared with the results in N_2_, indicating that the exothermic behavior is affected by SF_6_ decomposition.

Decomposition of SF_6_ is exacerbated with temperature increasing, and more free S and F atoms exist in the reaction gas to form a large amount of sulfides and fluorides resulting in increase of the exothermic peak area. It also confirms the conclusion that the smaller volume fraction of SF_6_ in the mixture would result in lower thermal stability, although it proved that 20% SF_6_/N_2_ has better dielectric property than 40% SF_6_/N_2_ [16]. Better dielectric property of 20% SF_6_/N_2_ cannot guarantee a better thermal property.

### 3.3. Effect of Epoxy Resin on the Decomposition Components of SF_6_/N_2_ Gas Mixture

In this study, 20% SF_6_/N_2_, 30% SF_6_/N_2_, and 40% SF_6_/N_2_ gas mixtures were selected as experimental gases and the heating temperature was in the range of 200 °C–650 °C. In order to detect the formation temperature of characteristic components as accurately as possible, small heating increment of 2 °C/min was selected, and time interval for continuous gas collection was 15 min, meaning that temperature rose by 30 °C. The volume of collected gas was about 0.3 L and the gas component concentration was detected by GC/MS instrument quickly. The average component concentration in 15 min reflects the component formation rate.

#### 3.3.1. Variation of Characteristic Decomposition Components with Temperature

Seven SF_6_ decomposition characteristic gases including CO_2_, SO_2_, H_2_S, SOF_2_, SO_2_F_2_, CF_4_, and CS_2_ were detected in our study. If the measured characteristic gas concentration is less than 0.05 ppm, it is considered to be below the detection limit of the instrument, meaning the gas is not generated. Judging by the above principle, in the entire temperature range of the experiment, no CS_2_ or SO_2_F_2_ was detected. Therefore, five gases including CO_2_, SO_2_, H_2_S, SOF_2_, and CF_4_ were selected as the characteristic decomposition components of overheating failure on epoxy resin surface.

**Figure 3:**
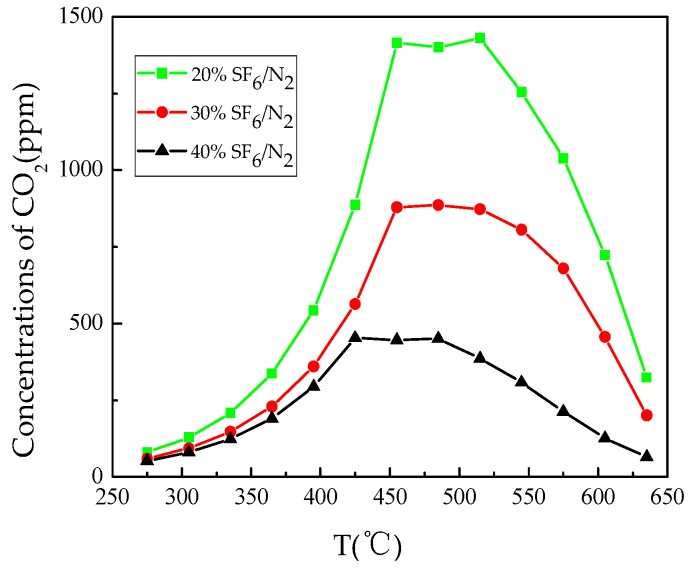
Formation of CO_2_ with temperature.

Figure 3 shows the formation of CO_2_ with temperature. It can be seen that the initial temperature of CO_2_ formation was always 275 °C under three gas mixtures with different proportions and the rate of CO_2_ formation increased before 450 °C and the generation rate of CO_2_ was fastest in the 20% SF_6_/N_2_. The formation rate of CO_2_ tended to become constant during a small temperature range after 450 °C. Finally, the CO_2_ formation rates of all three gases began to decrease from 515 °C.

**Figure 4:**
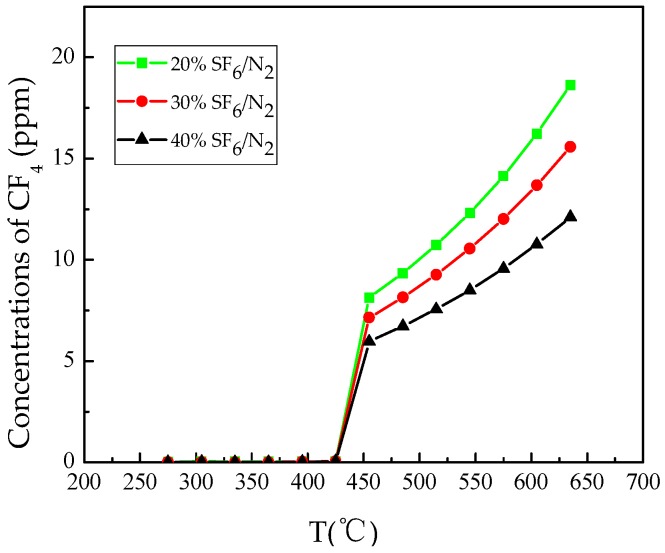
Formation of CF_4_ with temperature.

Figure 4 shows the pattern of CF_4_ generation with temperature. The initial temperature of CF_4_ formation was 455 °C under three different ratios of gas mixtures, and the formation rate of CF_4_ increased exponentially with the increase of temperature. The relationship of the initial formation concentration shows in the order: 20% SF_6_/N_2_ > 30% SF_6_/N_2_ > 40% SF_6_/N_2_, that is to say, CF_4_ has fastest formation rate in the 20% SF_6_/N_2_ mixture.

The initial formation temperature of CO_2_ and CF_4_ in the presence of epoxy resin in mixed gas is the same as the results in pure SF_6_ [2]. By observing the gas formation of CO_2_ and CF_4_, it can be concluded that the formation rate of CO_2_ became constant when CF_4_ started to be generated, and the rate of CO_2_ formation started to decrease versus the increase of CF_4_ generation rate, which is likely to be caused by the fact that C atom preferentially binds with F atom above 450 °C, which affects the formation rate of CO_2_.

**Figure 5:**
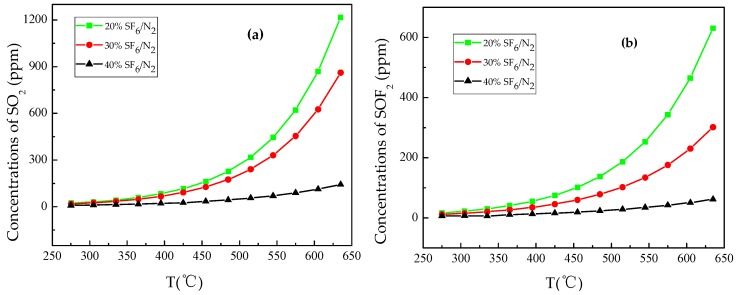
Formation of SO_2_ and SOF_2_ with temperature (**a**) SO_2_; (**b**) SOF_2_.

Figure 5 shows the formation of SO_2_ and SOF_2_ with temperature respectively. The initial formation temperature of SO_2_ and SOF_2_ was 275 °C under three gas mixtures with different ratios. The formation rates of SO_2_ and SOF_2_ increased exponentially with the increase of temperature, and the relationship of the initial formation concentration shows in the order: 20% SF_6_/N_2_ > 30% SF_6_/N_2_ > 40% SF_6_/N_2_. With temperature increasing, the formation rate of SO_2_ was bigger than that of SOF_2_.

Compared with the decomposition of epoxy resin in SF_6_ atmosphere, the formation temperature of SO_2_ and SOF_2_ in the presence of epoxy resin is lower [2]. SOF_2_ reacts with H_2_O to form SO_2_. Therefore, it can be seen that the formation of SO_2_ is greatly affected by H_2_O. During the decomposition of epoxy resin, more H_2_O is produced because of the dehydration condensation during elimination reaction within the molecule, which objectively enhances the hydrolysis of SOF_2_ and the formation of SO_2_.

**Figure 6:**
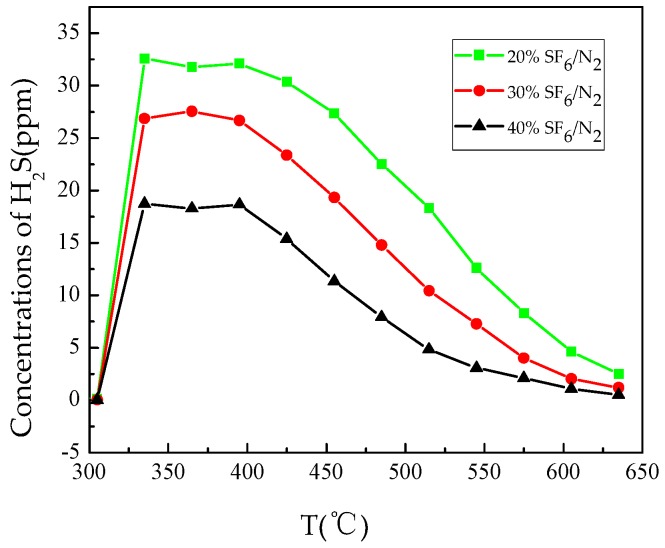
Formation of H_2_S with temperature.

Figure 6 shows the formation of H_2_S with temperature. The initial formation temperature of H_2_S in three gas mixtures with different ratios was about 335 °C. The H_2_S formation rate tended to be constant between 335 °C and 395 °C, and started to decrease above 425 °C. The formation of H_2_S and CF_4_ indicates that epoxy resin has entered the stage of rapid decomposition and weight loss. Early detection of this stage can effectively help avoid the serious consequences of further aggravation of overheating fault.

#### 3.3.2. Main Criteria for Determining Weight Loss of Epoxy Resins

It can be seen from the TG curves that the main weight loss temperature range of epoxy resin was 330 °C–470 °C. In this range, under SF_6_/N_2_ gas mixture conditions, main criteria for determining weight loss of epoxy resins can be concluded based on the concentration change of the characteristic components, such as H_2_S mainly generated in the range of 320 °C–350 °C while CF_4_ generated in the range of 440 °C–470 °C, the initial concentrations of H_2_S and CF_4_ had an obvious difference.

In order to obtain the standard for detecting the occurrence of an overheating fault, during heating samples of epoxy resin, concentrations of five characteristic components of CO_2_, SO_2_, H_2_S, SOF_2_, and CF_4_ were measured in the interval of 15 °C. Statistically the proportion of H_2_S and CF_4_ was used to determine whether the epoxy resin began to lose weight rapidly. When CF_4_ was not detected, the variation of H_2_S ratio was used as the criterion. Table 5 shows that the concentrations of H_2_S at different temperature range during the decomposition of epoxy resin under three gas mixtures. By the way, the concentrations represent the measured value at every interval rather than a cumulative value, reflecting formation rate of H_2_S. The formation rate of H_2_S decreased with temperature.

To obtain a mathematical principle for detecting the sharp weight-losing of epoxy resin, the concentrations of H_2_S in Table 5 should sum up. Because heating rate was 2 °C/min, the sum of concentrations from 320 °C to 440 °C was concentration in one hour. The total concentration of the five characteristic components generated in an hour is set as C(T), as shown in Table 6. C(H_2_S) represents the concentration of H_2_S. Before the occurrence of the CF_4_, the following criteria for determining the weight loss of epoxy resin can be obtained:

C(H_2_S)/C(T) > 0.01 (7)

On the other hand, CF_4_ began to form from 440 °C. Table 7 shows concentrations of CF_4_ formed at different temperature range during the decomposition of epoxy resin under three gas mixtures. The formation rate of CF_4_ increases exponentially with the increase of temperature.

The appearance of CF_4_ indicates that the rapid weight loss of epoxy resin has come to the late stage, and serious overheating fault has occurred. Setting the total concentration of five characteristic components as C(T), and C(CF_4_) as the concentration of CF_4_, the following criteria can be obtained for determining the weight loss range of epoxy resin.

C(CF_4_)/C(T) > 0.001 (8)

Epoxy resin in SF_6_-infused electrical equipment are designed to have high thermal and dielectric properties. To assess the operating condition of the sealed equipment based on the concentration of characteristic gases change would help find the overheating fault at an early stage. Equation (7) reflects the decomposition of epoxy resin at an early stage and Equation (8) represents the severe condition that epoxy resin enters sharp weight-losing stage. Once partial overheating faults happen on the surface of insulating material, usually it would be a lasting process to cumulate heat slowly so carbonization of epoxy resin in a small defect spot need to be analyzed both by Equations (7) and (8).

### 3.4. XPS Analysis

**Figure 7:**
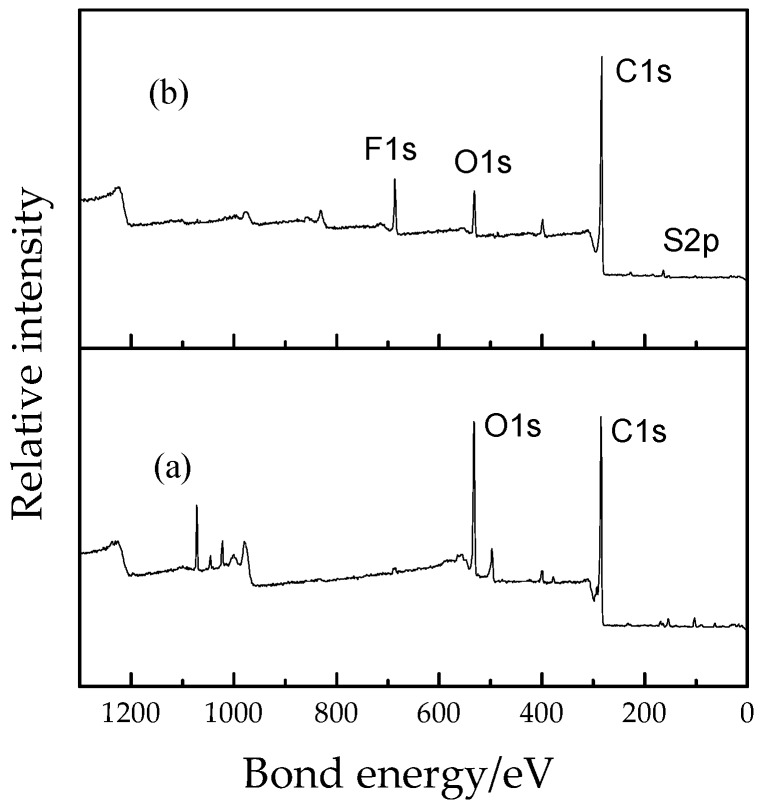
XPS spectrums of residues of pure epoxy after decomposition (**a**) in air (**b**) in SF_6_ heated after 500 °C.

Figure 7 shows XPS spectrum of burnt residue of epoxy resin in air and in SF_6_ atmosphere. Figure 7a shows the XPS spectrum of the epoxy resin in air. It can be seen that the peak intensity of the oxygen element and the carbon element are relatively high.

In Figure 7b, the elemental peaks of F1s and S2p are found in the residue of the epoxy resin after pyrolysis in SF_6_, indicating that some of the fluoride and sulfide are present in the residue. It is highly probable that fluorine is adsorbed in the form of CF_4_ by strong hydrogen bonds formed by carbonization of epoxy resin and the specific molecular structure deserves further study. The content of sulfur element is lower than that of fluorine element, indicating that the sulfur element interacts with the epoxy resin as long as it is released as SO_2_ gas. The chemical reaction mechanism of epoxy resin and SF_6_ gas also deserves further study.

### 3.5. Simulation of Decomposition Mechanism of Epoxy Resin

**Figure 8:**
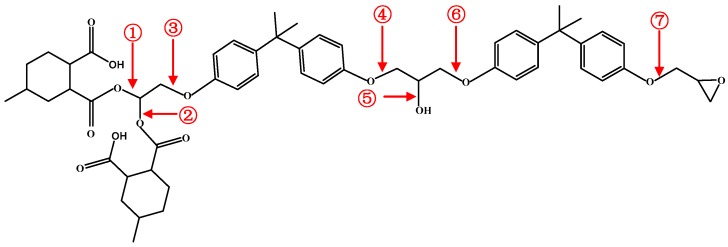
Single cured epoxy resin molecule.

As to the intrinsic decomposition mechanism of epoxy resin at high temperature, the simulation analysis was carried out by using ReacFF force field in Materials Studio software to study the formation of small molecular gases such as CO_2_ and H_2_O. Firstly, the molecular model of the bisphenol A epoxy resin after curing was constructed as shown in Figure 8. In the figure, ①, ②, ③, ④, ⑤, ⑥, and ⑦ represent C-O bonds at different positions respectively. The unit cell models of 15 epoxy resin molecules were created by using the Construction function in the tool of Amorphous Cell.

**Figure 9:**
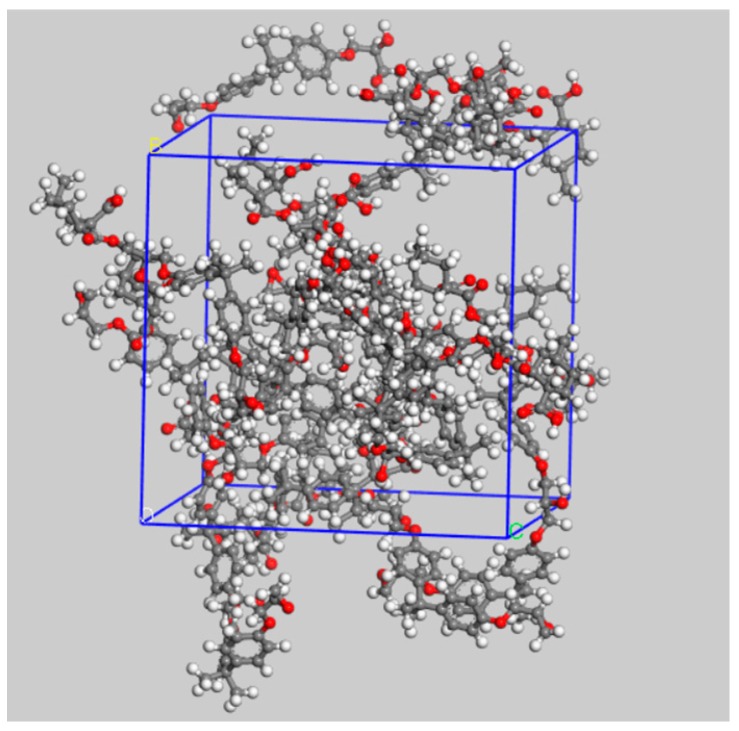
A unit cell model for epoxy resin.

The steps are summarized as follows: Single epoxy resin molecule shown in Figure 8 was constructed, and an initial density of 0.5 g/cm^3^ was applied. After 300 ps NPT ensemble simulation followed by 1000 ps structural optimization, the final unit cell model with the density of 1.14 g/cm^3^ was obtained, which is shown in Figure 9. After that, the ReaxFF force field was employed to simulate the decomposition process of the unit cell model at a maximum temperature of 1300 K during local discharge.

**Figure 10:**
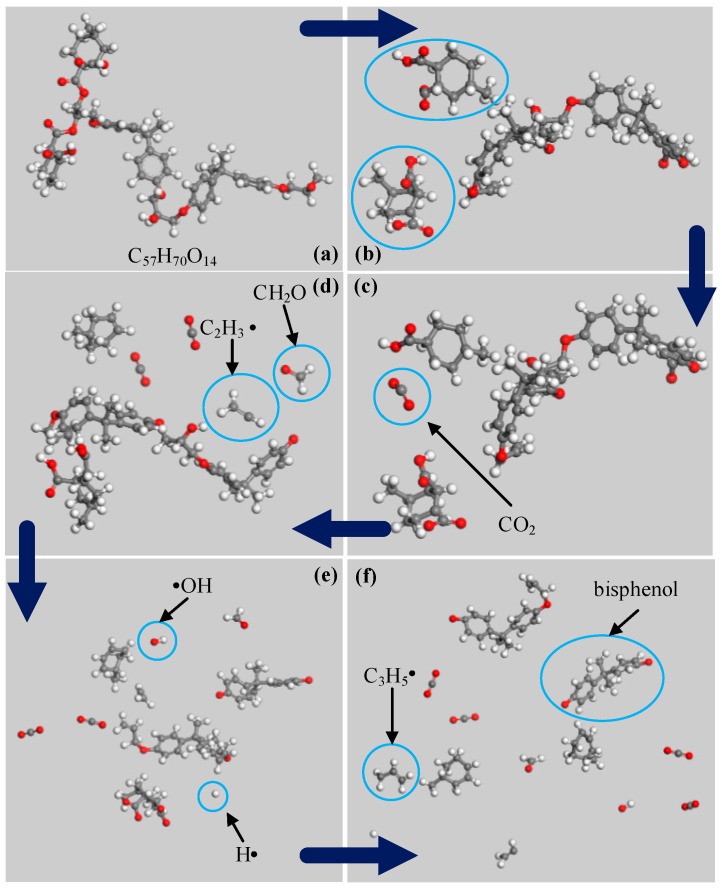
Schematic diagram of simulated bond-breaking process.

Figure 10 is a schematic diagram of the simulated bond breaking process. Figure 10a represents an epoxy resin molecule with the chemical formula of C_57_H_70_O_14_. The decomposition of epoxy resin starts with the cleavage of the C-O bond at locations ① and ② in Figure 8, which have the lowest activation energy, as shown in Figure 10b. After the cleavage the CO_2_ molecule is directly generated, as shown in Figure 10c. Following that, the C-O bond at location ⑦ in Figure 8 is cleaved, and ethylene radicals and CH_2_O are formed, as shown in Figure 10d. The C-O bonds at locations ⑤ and ⑥ in Figure 8 are cleaved to form free hydroxyl groups, which generate H_2_O when encountering highly active H ions, as shown in Figure 10e. Finally, propylene radicals and active bisphenol ions appear in Figure 10f.

**Figure 11:**
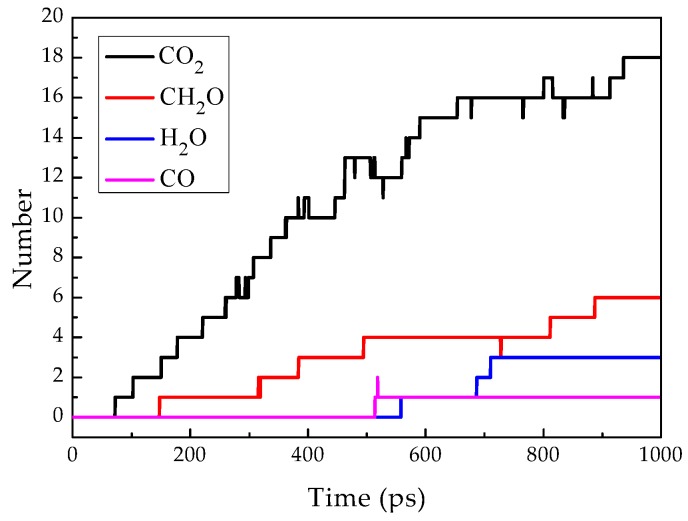
Theoretical byproduct numbers change during decomposition of epoxy resin cell over time.

Figure 11 shows the decomposition products of the epoxy resin unit cell model as a function of time. It can be seen that CO_2_ appears around 70 ps, followed by CH_2_O, and finally H_2_O. The concentration of CO_2_ is higher than that of H_2_O. CO_2_ mainly comes from the cleavage of the ester bond connecting the epoxy group, and H_2_O mainly comes from the elimination reaction of macromolecular ions and the dehydration condensation reaction between molecules.

It is conventionally stipulated that the maximum concentration of H_2_O in the main air chamber of the SF_6_ gas-insulated equipment to be put into operation should not exceed 500 ppm and the air content should not exceed 1%. Therefore, when there is an early latent insulation fault inside the equipment, the content of each component produced by the decomposition of SF_6_ will increase with the duration of discharge and overheating, and then the rate of increase will gradually slow down, and finally will reach a state of dynamic equilibrium until further increase happens due to aggravated fault. The presence of organic insulating material is one of the factors that accelerate the deterioration. At high temperature, SF_6_ is decomposed into low fluorides such as SF_4_ and SF_5_, which then react with H_2_O and O_2_ molecules. The decomposition process is shown in Equations 9–15.

(9) $${\mathrm{SF}}_{2}{+O}_{2}\to {\mathrm{SO}}_{2}F_{2}$$

(10) $${\mathrm{SF}}_{4}{+H}_{2}O\to {\mathrm{SOF}}_{2}+2\mathrm{HF}$$

(11) $${2\mathrm{SF}}_{5}{+H}_{2}O\to {2\mathrm{SOF}}_{4}+2\mathrm{HF}$$

(12) $${\mathrm{SOF}}_{4}{+H}_{2}O\to {\mathrm{SO}}_{2}F_{2}+2\mathrm{HF}$$

(13) $${\mathrm{SOF}}_{2}{+H}_{2}O\to {\mathrm{SO}}_{2}+2\mathrm{HF}$$

(14) $${\mathrm{SF}}_{6}\to S^{*}+6F$$

(15) $$S^{*}+2H\to H_{2}S$$

The H_2_O molecules generated by epoxy resin due to heating will react with SF_6_ to form SO_2_F_2_, SOF_2_, SO_2_ and other gases, which greatly affects the concentrations of decomposition components of SF_6_. By detecting the changes in concentration of each type of gas and comparing that with the thermal decomposition components of pure SF_6_, the rule of concentration change of the characteristic gases of the SF_6_ thermal decomposition components in the presence of epoxy resin can be summarized, and a basic method for judging whether there is a thermal decomposition fault of the epoxy resin in the electrical equipment can be proposed.

## 4. Conclusions

The thermal decomposition characteristics of epoxy resin in SF_6_/N_2_ mixture were studied. The concentration of characteristic decomposition components was detected. The following conclusions are drawn:

1. The TG curve shows that the main weight loss range of epoxy resin is 330 °C–470 °C. The degree of weight loss follows: N_2_ > 20% SF_6_/N_2_ > 30% SF_6_/N_2_ > 40% SF_6_/N_2_ > SF_6_. The DSC curves show that the decomposition of epoxy resin under SF_6_ condition is a series of complex chemical reactions. Epoxy resin decomposition in the 20% SF_6_/N_2_ is more severe than in 40% SF_6_/N_2_.
2. During heating from 200 °C to 650 °C, the five gases of CO_2_, SO_2_, H_2_S, SOF_2_, and CF_4_ are selected as the characteristic decomposition components of SF_6_. CO_2_, SO_2_ and SOF_2_ are all formed at 275 °C and the formation rate increases exponentially, but the formation rate of CO_2_ gradually decreases after CF_4_ has been generated. The formation rate of SO_2_ is higher than that of SOF_2_. The reason is that the decomposition of epoxy resin produces H_2_O, promoting hydrolysis of SOF_2_.
3. When epoxy resin was heated in the gas mixture, the initial generation temperature of H_2_S was lower than in the pure SF_6_, and CF_4_ generation rate also relatively increased. Concentration change of H_2_S and CF_4_ can be used as the criteria for judging the sudden weight loss caused by overheating fault happening on the surface of epoxy resin. 20% SF_6_/N_2_, 30% SF_6_/N_2_, and 40% SF_6_/N_2_ share the same judging standard: When there is no CF_4_ generation, H_2_S is used as the criterion depicted as follow: C(H_2_S)/C(T) > 0.01; when CF_4_ is generated, CF_4_ is used as the criterion depicted as follow: C(CF_4_)/C(T) > 0.001, among which C(T) represents the total decomposition gas concentration.
4. Thermal decomposition process of epoxy resin was simulated by the ReaxFF force field to reveal basic chemical reactions in terms of bond-breaking order, which further verified that CO_2_ and H_2_O produced during thermal decomposition of epoxy resin can intensify degradation of SF_6_ dielectric property. To judge the operation situation of SF_6_-infused electrical equipment, the problem of overheating faults involving epoxy resin decomposition should draw more attention.

## Figures and Tables

**Table 1:** Experimental gas densities at 0 °C under standard atmospheric pressure.

Type of Gas	SF_6_	N_2_	20% SF_6_/N_2_	30% SF_6_/N_2_	40% SF_6_/N_2_
**Density/(kg/m^3^)**	6.5200	1.2506	2.3046	2.8316	3.3586

**Table 2:** Specific heat capacity of experimental gases at constant pressure at 25 °C under standard atmospheric pressure.

Type of Gas	SF_6_	N_2_	20% SF_6_/N_2_	30% SF_6_/N_2_	40% SF_6_/N_2_
Specific Heat Capacity/(J/kg·K)	665.180	1040.000	827.893	781.055	748.910

**Table 3:** The calculated values of thermal conductivities at 0 °C under standard atmospheric pressure.

Types of Gas	SF_6_	N_2_	20% SF_6_/N_2_	30% SF_6_/N_2_	40% SF_6_/N_2_
Thermal Conductivity/(W/m·K)	0.01206	0.02598	0.02378	0.02256	0.02129

**Table 4:** Experimental gas exothermic peak energy at 330 °C–470 °C.

Types of Gas	SF_6_	N_2_	20% SF_6_/N_2_	30% SF_6_/N_2_	40% SF_6_/N_2_
Exothermic Peak Energy/mJ	9359.28	6718.17	11,648.33	10,520.45	9587.56

**Table 5:** Concentrations of H_2_S at different temperature range in the interval of 15 °C (ppm).

Unit/%	320 °C	335 °C	350 °C	365 °C	380 °C	395 °C	410 °C	425 °C	440 °C
20% SF_6_/N_2_	9.44	10.12	9.01	7.63	5.38	4.97	3.94	2.80	1.24
30% SF_6_/N_2_	0	9.32	8.96	7.82	5.01	4.11	2.88	2.63	0.99
40% SF_6_/N_2_	0	10.10	9.22	7.77	5.89	4.82	4.12	3.62	2.09

**Table 6:** Total concentration of five characteristic gases at different temperature range in the interval of 15 °C (ppm).

Unit/%	350 °C	365 °C	380 °C	395 °C	410 °C	425 °C	440 °C	455 °C	470 °C
20% SF_6_/N_2_	1037.81	1440.82	1673.29	2152.99	2435.98	3367.67	4359.82	5167.27	5557.31
30% SF_6_/N_2_	733.95	908.33	1023.89	1332.36	1241.07	1996.00	2112.52	2985.67	3811.16
40% SF_6_/N_2_	527.16	727.37	772.42	1051.19	1113.77	1554.41	1493.30	1596.54	2095.89

**Table 7:** Concentrations of CF_4_ at different temperature range in the interval of 15 °C (ppm).

Unit/%	440 °C	455 °C	470 °C
20% SF_6_/N_2_	0.10	0.10	0.11
30% SF_6_/N_2_	0.18	0.23	0.24
40% SF_6_/N_2_	0.28	0.31	0.32
